# Estimated incidence of pertussis in people aged <50 years in the United States

**DOI:** 10.1080/21645515.2016.1186313

**Published:** 2016-05-31

**Authors:** Chi-Chang Chen, Catherine Balderston McGuiness, Girishanthy Krishnarajah, Christopher M. Blanchette, Yuanyuan Wang, Kainan Sun, Philip O. Buck

**Affiliations:** a IMS Health, Plymouth Meeting, PA, USA; b GSK, Philadelphia, PA, USA; c University of North Carolina, Charlotte, NC, USA

**Keywords:** incidence, pertussis, underreporting, US, whooping cough

## Abstract

The introduction of pertussis vaccination in the United States (US) in the 1940s has greatly reduced its burden. However, the incidence of pertussis is difficult to quantify, as many cases are not laboratory-confirmed or reported, particularly in adults. This study estimated pertussis incidence in a commercially insured US population aged <50 years. Data were extracted from IMS' PharMetrics Plus claims database for patients with a diagnosis of pertussis or cough illness using International Classification of Diseases (ICD-9) codes, a commercial outpatient laboratory database for patients with a pertussis laboratory test, and the Centers for Disease Control influenza surveillance database. US national pertussis incidence was projected using 3 methods: (1) diagnosed pertussis, defined as a claim for pertussis (ICD-9 033.0, 033.9, 484.3) during 2008–2013; (2) based on proxy pertussis predictive logistic regression models; (3) using the fraction of cough illness (ICD-9 033.0, 033.9, 484.3, 786.2, 466.0, 466.1, 487.1) attributed to laboratory-confirmed pertussis, estimated by time series linear regression models. Method 1 gave a projected annual incidence of diagnosed pertussis of 9/100,000, which was highest in those aged <1 year. Method 2 gave an average annual projected incidence of 21/100,000. Method 3 gave an overall regression-estimated weighted annual incidence of pertussis of 649/100,000, approximately 58–93 times higher than method 1 depending on the year. These estimations, which are consistent with considerable underreporting of pertussis in people aged <50 years and provide further evidence that the majority of cases go undetected, especially with increasing age, may aid in the development of public health programs to reduce pertussis burden.

## Introduction

Pertussis, also known as whooping cough, is a respiratory illness caused by infection with the bacterium *Bordetella pertussis*. Typical symptoms – generally seen in children – include paroxysms of uncontrollable violent coughing ending in the characteristic inspiratory whoop, frequently followed by vomiting.^1^ Older individuals often do not present with these typical symptoms, and may only have a persistent cough.^1^ The infection is highly contagious, particularly in the early stages of illness.^1^ Before the availability of pertussis vaccines (i.e. before the 1940s), pertussis was a major cause of morbidity and mortality in infants and children in the United States (US).^2^ After the introduction and widespread uptake of diphtheria-tetanus-pertussis (DTP) vaccine in the 1940s, pertussis incidence, as reported by national surveillance data, declined sharply in the US, reaching a low in 1976.^2^ However, the reported pertussis incidence has increased overall in the US since the 1980s, with peaks every 2–5 year.^3^ In 2013, there were 28,639 cases of reported pertussis in the US and 13 pertussis-related deaths.^4^

The increase in pertussis^3^ has occurred despite high rates of childhood vaccination.^5^ Clinical pertussis infection in childhood does not confer lifelong immunity against the disease,^6^ and neither does pediatric vaccination.^7^ Further, vaccine effectiveness has been reported to wane each year after the fifth dose (at age 4–6 years)^8^ of diphtheria, tetanus and acellular pertussis (DTaP)^9,10^ or tetanus, diphtheria and acellular pertussis (Tdap)^11,12^ vaccines in the US. Older children may thus become susceptible to pertussis infection after waning of the immunity conferred by vaccination before they reach the recommended age for the Tdap adolescent booster (11–12 years)^8^. Further, it seems likely that vaccinated adolescents and adults will also not have long-term protection due to waning immunity.

Although pertussis mortality is concentrated in young infants (12/13 reported pertussis deaths in the US in 2013 occurred in infants aged <3 months),^4^ the burden of pertussis morbidity in adolescents and adults is substantial. Pertussis causes disruption of sleep and daily activities and impairs quality of life.^13^ In a study in adults aged ≥20 years with culture-verified pertussis in Sweden, 87 of 134 patients in employment had to stay away from work, usually for 2–4 weeks.^6^ In Canada, adolescents with pertussis missed a mean of 5 days from school, and adults lost a mean of 7 work days.^14^ Furthermore, adults with pertussis may also be an important source of disease spread,^6^ including transmission to susceptible infants who are too young to be vaccinated.^15^

The incidence of pertussis in adolescents and adults is very difficult to quantify. Many cases are not recognized or diagnosed, as the symptoms may be misdiagnosed as other respiratory illnesses, infected individuals may not seek medical care, and pertussis may not be considered as a diagnosis in adults.^15-17^ Pertussis cases reported by national surveillance systems may therefore be an underestimate of the number of pertussis cases in adults. A retrospective claims data study of older adults diagnosed with pertussis in the US found that the estimated incidence in people aged 65 years or more exceeded the incidence reported by the national surveillance data in each of the 5 years of the study.^18^ A clinical trial in the US estimated the annual incidence of laboratory-confirmed pertussis at 370 per 100,000 in people aged 15–65 years over the period 1997–1999, which would equate to almost one million cases per year if extrapolated to the US population.^19^ This is far in excess of the number of cases reported to national surveillance.^4^

Better understanding of the full burden of pertussis illness will help to support the development of strategies (e.g., vaccination of additional age and/or risk groups) to control the disease. The objective of the present analysis was to estimate the incidence of pertussis in a commercially insured US population aged <50 years. Three different methods were used in order to explore the possible range of pertussis incidence: (1) claims for pertussis diagnosed using International Classification of Diseases, Ninth Revision, Clinical Modification (ICD-9) codes; (2) proxy pertussis predictive logistic regression models (based on symptoms that could indicate undiagnosed pertussis); and (3) the fraction of cough illness statistically attributed to laboratory-confirmed pertussis estimated by time series linear regression models.

## Results

### Sample size, demographic and clinical characteristics

Table 1 shows the number of patients with ICD-9-diagnosed pertussis and the number of patients with cough illness meeting the study criteria who formed the study samples for methods 1 and 3 by year. A total of 5,163 patients with pertussis test results (1,581 tested positive, 3,582 tested negative) across the 6 years formed the sample for the proxy pertussis analysis (method 2).

**Table 1.** Sample sizes by year for methods 1 and 3.

	**Number of patients**	
**Year**	**ICD-9 diagnosed pertussis (method 1)**	**ICD-9 diagnosed pertussis or cough illness (method 3)**
2008	1,295	2,685,986
2009	1,411	3,335,514
2010	1,891	2,302,043
2011	1,241	2,540,936
2012	2,605	2,508,737
2013	1,417	2,383,060

ICD, International Classification of Diseases

Table 2 shows the demographic characteristics of patients included in each of the 3 analyses. For the analyses with multi-year samples, the demographic characteristics are shown for the most recent year (2013). The samples had broadly similar demographic characteristics, except that the cough illness sample (method 3) and the cases in the proxy pertussis sample (method 2) were older than the ICD-9-diagnosed pertussis sample (method 1).

**Table 2.** Demographic characteristics of patients included in each analysis.

		**Proxy pertussis logistic regression sample, 2008–2013 (method 2)**		
**Characteristic**	**ICD-9 diagnosed pertussis, 2013 (n = 1,417) (method 1)**	**Cases^a^ (n = 1,581)**	**Controls^b^ (n = 3,582)**	**ICD-9 diagnosed pertussis or cough illness, 2013 (n = 2,383,060) (method 3)**
**Age groups, n (%)**				
<1 year	159 (11.2%)	62 (3.9%)	484 (13.5%)	31,200 (1.3%)
0–3 months	107 (67.3%)	30 (48.4%)	263 (54.3%)	13,548 (43.4%)
4–6 months	40 (25.2%)	27 (43.5%)	165 (34.1%)	10,228 (32.8%)
7–9 months	11 (6.9%)	4 (6.5%)	52 (10.7%)	5,903 (18.9%)
10–11 months	1 (0.6%)	1 (1.6%)	4 (0.8%)	1,521 (4.9%)
1–6 years	255 (18.0%)	204 (12.9%)	1,004 (28.0%)	449,250 (18.9%)
7–10 years	161 (11.4%)	160 (10.1%)	437 (12.2%)	210,437 (8.8%)
11–18 years	328 (23.1%)	326 (20.6%)	628 (17.5%)	322,159 (13.5%)
19–49 years	514 (36.3%)	829 (52.4%)	1,029 (28.7%)	1,370,014 (57.5%)
**Gender, n (%)**				
Female	805 (56.8%)	950 (60.1%)	1,998 (55.8%)	1,297,788 (54.5%)
Male	612 (43.2%)	631 (39.9%)	1,584 (44.2%)	1,085,272 (45.5%)
**Geographic region, n (%)**				
Northeast	344 (24.3%)	338 (21.4%)	841 (23.5%)	468,803 (19.7%)
Midwest	461 (32.5%)	270 (17.1%)	951 (26.5%)	586,996 (24.6%)
South	490 (34.6%)	827 (52.3%)	1,463 (40.8%)	1,177,128 (49.4%)
West	122 (8.6%)	146 (9.2%)	327 (9.1%)	150,133 (6.3%)
**Payer Type, n (%)**				
Commercial	889 (62.7%)	864 (54.6%)	1,932 (53.9%)	1,488,461 (62.5%)
Medicaid	60 (4.2%)	47 (3.0%)	181 (5.1%)	91,921 (3.9%)
Self-insured	451 (31.8%)	637 (40.3%)	1,395 (38.9%)	786,922 (33.0%)
Medicare Risk / Other/Unknown	17 (1.2%)	33 (2.1%)	74 (2.1%)	15,756 (0.7%)

a Cases had a confirmed pertussis test result that was positive

b Controls had a confirmed pertussis test result that was negative

ICD, International Classification of Diseases

Table 3 shows the main comorbidities recorded for the patients with ICD-9-diagnosed pertussis (method 1), together with the index diagnoses. The patients were generally in good health, with a median Charlson comorbidity index score of 0 in the 536 patients aged 18 or over. The most common comorbid conditions were upper and lower respiratory tract infection. Over two-thirds of the index pertussis diagnoses were coded as ICD-9 033.9 (whooping cough, unspecified organism).

**Table 3.** Co-morbid conditions and index diagnoses in the sample of patients with ICD-9-diagnosed pertussis, 2013 (n = 1,417) (method 1).

	Number	%
**Most frequently observed comorbid conditions of interest in the pre-index period**		
**Patients aged < 18 years**	**881**	
Upper respiratory tract infection	415	47.1%
Lower respiratory tract infection	140	15.9%
Asthma	133	15.1%
Allergy	114	12.9%
Congenital anomalies	45	5.1%
Symptoms concerning nutrition, metabolism and development	43	4.9%
Osteoarthritis/Spinal conditions	17	1.9%
Sleep disorders	8	0.9%
Cardiac arrhythmia	8	0.9%
Epilepsy/Seizure disorder	6	0.7%
**Patients ≥ 18 years**	**536**	
Upper respiratory tract infection	175	32.6%
Lower respiratory tract infection	134	25.0%
Osteoarthritis/Spinal conditions	76	14.2%
Allergy	58	10.8%
Asthma	58	10.8%
Hypertension	49	9.1%
Depression	44	8.2%
Diabetes	23	4.3%
Obesity	22	4.1%
Smoking or history of smoking	22	4.1%
**Index diagnosis (ICD-9 code)**		
033.0 (whooping cough due to Bordetella pertussis)	433	30.6%
033.9 (whooping cough, unspecified organism)	969	68.4%
484.3 (pneumonia in whooping cough)	15	1.1%

ICD, International Classification of Diseases

### Projected incidence based on ICD-9-diagnosed pertussis (method 1)

**Figure 1.**
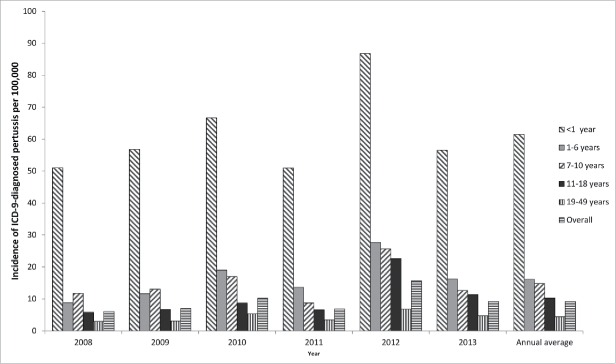
Projected national incidence (per 100,000) of ICD-9-diagnosed pertussis by year and age group (method 1). ICD, International Classification of Diseases.

The projected national annual pertussis incidence based on ICD-9-diagnosed pertussis cases is shown by age group and year in Figure 1 and by region, age group and year in Table 4. Across all years, age groups and regions, the overall weighted annual estimated incidence rate was 9 per 100,000 population. Incidence decreased with increasing age, and varied year-by-year with a peak in 2012 (Fig. 1).

**Table 4.** Projected incidence per 100,000 of pertussis based on ICD-9-diagnosed pertussis cases, by age group, year and region (method 1).

	**6-year incidence rate (2008–2013)**	**2008**	**2009**	**2010**	**2011**	**2012**	**2013**	**Weighted annual incidence rate**
**Overall**	**55**	**6**	**7**	**10**	**7**	**16**	**9**	**9**
**By Age Group and Region**								
<1 year, Northeast	408	62	62	52	47	142	45	68
<1 year, Midwest/West	372	45	44	80	66	90	51	63
<1 year, South	344	54	70	56	36	60	69	58
1 – 6 years, Northeast	130	19	14	21	14	47	14	22
1 – 6 years, Midwest/West	113	8	11	26	19	31	18	19
1 – 6 years, South	63	5	11	10	6	15	15	10
7 – 10 years, Northeast	122	14	12	28	12	45	11	20
7 – 10 years, Midwest/West	96	11	13	20	11	28	13	16
7 – 10 years, South	64	12	14	8	5	13	13	11
11 – 18 years, Northeast	89	9	6	12	10	38	14	15
11 – 18 years, Midwest/West	73	6	7	11	8	27	13	12
11 – 18 years, South	35	4	6	4	3	9	8	6
19 – 49 years, Northeast	29	4	3	5	4	9	4	5
19 – 49 years, Midwest/West	31	3	3	7	4	8	5	5
19 – 49 years, South	19	2	3	3	2	4	4	3

ICD, International Classification of Diseases

Comparison of medical claims data and laboratory data indicated that the 3 pertussis ICD-9 diagnosis codes had low sensitivity (30.4%) and high specificity (94.0%), with a positive predictive value of 68.2%. The low sensitivity implies that many people with a laboratory-confirmed pertussis infection did not have one of these ICD-9 pertussis diagnosis codes, while the high specificity indicates that most patients who had one of these diagnoses truly had a pertussis infection.

### Projected incidence based on proxy pertussis (method 2)

The projected annual national pertussis incidence based on the proxy pertussis logistic regression model is shown by age group and region in Table 5. The overall average estimated annual incidence rate was 21 per 100,000, higher than the incidence based on ICD-9-diagnosed pertussis. Incidence was highest in the groups aged 1–6 and <1 year and then decreased with increasing age.

**Table 5.** Projected incidence of pertussis based on proxy pertussis logistic regression, by age group and region (method 2).

		**6-year incidence rate (2008–2013) per 100,000**			**Average annual incidence per 100,000**
**Age group**	**Region**	**Point estimate**	**Lower 95% CI**	**Upper 95% CI**	**Point estimate**
**Overall**		**130**	**125**	**135**	**21**
<1 y	National	449	433	466	72
<1 year	Northeast	351	334	368	56
<1 year	Midwest/West	297	284	310	47
<1 year	South	678	658	699	110
1–6 y	National	572	554	591	92
1–6 years	Northeast	680	654	706	109
1–6 years	Midwest/West	334	322	347	54
1–6 years	South	818	796	839	133
7–10 y	National	164	155	172	26
7–10 years	Northeast	342	326	358	54
7–10 years	Midwest/West	111	105	117	18
7–10 years	South	146	138	154	23
11–18 y	National	93	88	97	15
11–18 years	Northeast	158	151	165	25
11–18 years	Midwest/West	75	72	79	12
11–18 years	South	81	78	85	13
19–49 y	National	21	20	22	3
19–49 years	Northeast	20	18	21	3
19–49 years	Midwest/West	12	11	12	2
19–49 years	South	33	32	35	5

CI, confidence interval

The model sensitivity decreased with increasing age, from 48.8% in the group aged <1 year to 24.6% in the group aged 19–49 years, and the model specificity was over 90% in all age groups (ranging from 91.1% in the group aged 1–6 years to 95.1% in the group aged 19–49 years).

### Projected incidence based on cough illness attributed to pertussis (method 3)

The final regression models included only laboratory-confirmed pertussis and influenza as pathogen predictors. The coefficients associated with respiratory syncytial virus (RSV) were negative and thus were removed from the model. The model-predicted cough illness estimates fit the observed data well. However, much of the variance was either unattributed or attributed to influenza, pertussis accounted for less than 5% of the variance across the time period, and was not statistically significant in most models (i.e., all but the model for the Southern region). The estimated pertussis incidence from these regression models should therefore be interpreted with caution.

**Figure 2.**
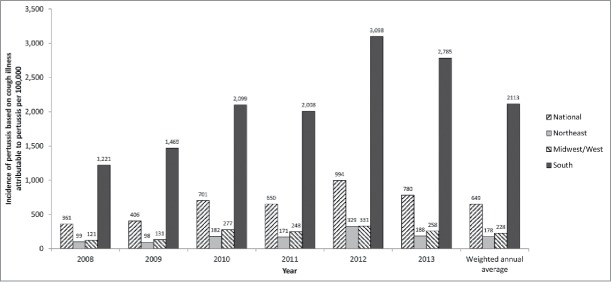
Projected national incidence (per 100,000) of pertussis based on the fraction of cough illness statistically attributable to pertussis by region and year (method 3).

The projected annual national pertussis incidence based on the fraction of cough illness statistically attributable to pertussis is shown by region and year in Figure 2. Estimates by age group were not available as no valid age-specific model could be obtained. Like the incidence estimated from ICD-9-diagnosed pertussis, the incidence varied year-by-year and peaked in 2012. Across all years and regions, the overall weighted annual incidence rate was 649 per 100,000 population, 72 times higher than the incidence based on ICD-9-diagnosed pertussis. This ratio varied from 58 to 93, depending on the year (Table 6).

**Table 6.** Ratio between estimated ICD-9-diagnosed pertussis incidence and estimated pertussis incidence based on the fraction of cough illness statistically attributable to pertussis by year.

**Year**	**ICD-9-diagnosed pertussis incidence (per 100,000) (method 1)**	**Pertussis incidence based on the fraction of cough illness statistically attributed to pertussis (per 100,000) (method 3)**	**Ratio of pertussis incidence based on statistically attributable cough illness to ICD-9-diagnosed pertussis (method 3/method 1)**
2008	6	361	60
2009	7	406	58
2010	10	701	70
2011	7	650	93
2012	16	994	62
2013	9	780	87
Overall	9	649	72

ICD, International Classification of Diseases

## Discussion

Before the introduction of pertussis vaccination in the 1940s, pertussis infection was a major cause of morbidity and mortality in children in the US, with more than 200,000 cases per year. Since pertussis vaccines became widely used, pertussis incidence in the US has decreased by more than 80% compared with the pre-vaccine era.^20^ However, pertussis incidence is challenging to measure, as many cases are not recognized or diagnosed, particularly in adults. By exploring 3 different methods for projecting pertussis incidence in a commercially insured US population, the present study should help to improve understanding of the potential range of the pertussis burden in people aged <50 years. To our knowledge, this is the first study to attempt this in this age group. A previous study using some of the same methods has estimated pertussis incidence in the US in mature and elderly adults (people aged ≥50 years).^21^

The three approaches resulted in a wide range of estimates for the projected national annual incidence of pertussis in the US over the period 2008–2013. The most conservative approach (method 1), using data from medical insurance claims with an ICD-9 code for pertussis, estimated the overall weighted annual incidence rate at 9 per 100,000 population. This is still likely to be an underestimate, as it did not include patients who tested positive for pertussis but did not have a medical claim with a pertussis ICD-9 code, nor does it include patients that were misdiagnosed or did not seek medical attention. The low sensitivity of the ICD-9 pertussis codes found in the present study indicates that many patients with laboratory-confirmed pertussis infection were not coded as such.

Method 2 did not rely on the presence of a pertussis ICD-9 code, but instead identified medical events that could be a proxy for laboratory-confirmed pertussis and used these data in a logistic regression model to estimate the fraction of patients that were pertussis cases. This proxy method estimated the overall average annual incidence rate at 21 per 100,000 across the study period, more than double the estimate obtained using ICD-9-diagnosed pertussis. However, the predictive models had low sensitivity, a limitation shared with the method using the ICD-9 codes alone. Thus, the proxy method may still underestimate the incidence of pertussis. We also note that this model predicted the highest pertussis incidence among those aged 1–6 years, which is not in line with Centers for Disease Control and Prevention (CDC) data,^22^ where those aged <1 year are most commonly affected. This may be because pertussis incidence was based on clinical events that could indicate undiagnosed pertussis. As coughs, upper respiratory tract infections, fever, etc. are so common among children aged 1–6 years, it is possible that the incidence of pertussis in this age group has been overestimated by this method.

Method 3 used pathogen data to estimate the fraction of cough illness statistically attributable to pertussis using logistic regression models. This approach has been widely used to estimate the burden of morbidity and mortality attributable to respiratory pathogens such as influenza and RSV in the US and Europe.^23-27^ In our study, this method produced the highest estimate of the overall weighted annual incidence rate of pertussis, at 649 per 100,000 population. This was 58 to 93 times higher than the incidence based on ICD-9-diagnosed pertussis, depending on the year. This method of attributing illness episodes among pathogens is dependent on the accuracy of the data available for the pathogens under consideration (RSV, influenza and pertussis). In the present analysis, the regression models were dominated by influenza data; the RSV coefficients were negative and therefore omitted from the final models, and the pertussis terms accounted for only a small fraction (<5%) of the model variance. This dominance of influenza may have compromised the ability of the model to attribute pertussis activity (i.e. positive pertussis laboratory test over time) to cough illness incidence, which may explain why pertussis model terms were not significant with the exception of the model for the Southern region resulting in substantial variation in incidence estimates between geographic regions using this method. Consequently, the results of method 3 should be interpreted with caution. It should also be noted that all of the 3 methods relied on medical claims data, and therefore could only identify medically-attended pertussis illness. Patients with pertussis who did not seek medical care would not have been captured in any of these analyses, and thus the present estimates may still be an underestimate of the burden of pertussis illness.

Other studies of pertussis incidence have also reported a wide range of results, consistent with the findings reported here. The US national surveillance system reported pertussis incidence rates for 2013 of 9.0 per 100,000 overall, ranging from 2.6 per 100,000 in people aged 20 y or more to 45.3 per 100,000 in infants aged 6–11 months and 160.3 in infants aged <6 months (approximately 100 per 100,000 in infants aged <1 year).^4^ The incidence estimates obtained using ICD-9-diagnosed pertussis in the present analysis, which yielded the lowest estimates of any of the 3 methods (9 per 100,000 overall, 4 per 100,000 in adults aged 19–49 years, and 61 per 100,000 in infants aged <1 year), were similar to these reported national incidence rates. A prospective study conducted in a managed care organization in Minnesota, US, in 1995–1996 enrolled adolescents and adults (aged 10–49 years) who presented with acute paroxysmal cough or a persistent cough lasting 7–34 days. Pertussis was defined as any positive laboratory test for pertussis (culture, polymerase chain reaction [PCR], or antibodies to pertussis toxin), combined with cough for ≥14 days and at least one of the following symptoms: paroxysmal cough, whoop or vomiting after coughing. The annual pertussis incidence was estimated at 507 per 100,000 person-years (95% confidence interval [CI] 307, 706).^28^ This is comparable with the estimated incidence in the present study based on the fraction of cough illness statistically attributable to pertussis, which was 649 per 100,000 in the population aged <50 years. The higher estimated incidence in the present study could reflect the more recent time period (2008–2013, compared with 1995–1996) and/or the younger age range (0–50 years, compared with 10–49 years). A cost-effectiveness analysis of pertussis vaccination in adults noted that the range of plausible pertussis incidence estimates in the literature is broad, and used a range of reported adult pertussis incidence from 10 per 100,000 to 500 per 100,000.^29^ This range is similar to the range of incidence results across the 3 methods in the current study (9 per 100,000 for ICD-9-diagnosed pertussis to 649 per 100,000 for incidence based on the fraction of cough illness statistically attributable to pertussis). More recently, a retrospective database study of insured adults aged ≥50 years seen by private healthcare practitioners in the US, using methods similar to the present analysis, found that the incidence of cough illness statistically attributable to pertussis was 42–105 times higher than the incidence of pertussis diagnosed by ICD-9 codes.^21^ These ratios are similar to the ratios of 58–93 reported in the present study.

The estimates of pertussis incidence from the present study are consistent with considerable underdiagnosis and underreporting of pertussis among people aged <50 years in the US; and highlight the need for improved preventive measures – such as increased vaccination – against pertussis. In addition, our finding that the most common comorbid conditions recorded in the database in the 90 days before the index date were upper and lower respiratory tract infection suggests that pertussis cases may initially receive a less specific diagnosis. These diagnoses may have been made pending confirmation of the pathogen from laboratory tests, or they may represent misdiagnosis.

The presence of a substantial incidence of pertussis indicates the need for improved control of pertussis infection, potentially by adding pertussis immunization recommendations for additional age and/or risk groups, or researching strategies to reduce waning immunity after vaccination. Improved control could not only reduce the direct burden of morbidity in infected individuals, but could also help to reduce transmission of pertussis infection to infants too young to be vaccinated, in whom pertussis can be severe or fatal. Booster vaccination of adolescents and adults could help to restore immunity levels after the effect of childhood pertussis vaccination has begun to wane. The US Advisory Committee on Immunization Practices (ACIP) guidelines recommend a single dose of Tdap for all children (preferred age 11–12 years) and adults aged 19 y or older who have not received Tdap vaccination.^8^ Several countries in Europe have also introduced booster pertussis vaccination for adolescents and adults.^15^ To reduce the risk of pertussis transmission to newborn infants, maternal immunization of pregnant women against pertussis is recommended by ACIP in the US,^8^ and various strategies for cocooning vaccination (vaccination of parents and/or other family contacts of newborn infants), have been proposed in Europe.^15^ However, it should be noted that not all cases of whooping cough are caused by *B. pertussis*, and that immunization with currently available vaccinations would not provide protection against whooping cough caused by other organisms.^30^

The study has a number of limitations. First, the study is based on an insurance claims database and therefore represents a commercially insured US population. The results may not generalize to US populations without medical insurance. Second, claims data are collected for the purposes of reimbursement and billing, and this may influence the recording of information. For example, practitioners may be reluctant to submit a claim with a pertussis code in the absence of laboratory confirmation, and if a diagnosis was initially submitted with a non-specific code pending test results it would not be changed to a pertussis code if test results subsequently confirmed pertussis unless a follow-up visit triggered an additional claim. Upper and lower respiratory tract infection pre-index diagnoses were the most common co-morbid conditions in the database sample, and it is likely that there were an unknown number of other such cases that were not followed by a pertussis diagnosis code and thus were not captured in the study. Third, the laboratory test database used in the study only collects data for tests performed in an outpatient setting. Fourth, the clinical and laboratory methods used to diagnose pertussis do not have 100% sensitivity or specificity, so some diagnoses may not be accurate. After the fourth week of cough, the amount of bacterial deoxyribonucleic acid diminishes rapidly and thus PCR tests may not detect pertussis accurately if the patient seeks treatment after this period.^21^ Fifth, as mentioned above, the incidence based on the fraction of cough illness statistically attributable to pertussis should be interpreted with caution, because the models could not be stratified by age and the pertussis terms in the regression models were not always statistically significant. Also, pertussis symptoms vary by age,^1^ further complicating this analysis. Lastly, there were more children in the ICD-9 pertussis (method 1) and proxy controls (method 2) than in the proxy cases (method 2) and ICD-9 cough (method 3) groups. This likely reflects the better diagnosis of pertussis among children, but we acknowledge that this may have affected the estimates obtained from these methods.

The wide variation between the estimated incidences of pertussis based on the 3 different methods emphasizes the challenges in estimating pertussis incidence. The range of incidence estimates presented in this analysis may give an indication of the likely upper and lower bounds of the incidence of pertussis, but further research will be needed to test the current findings and to refine the estimates further.

## Conclusion

The estimations of pertussis incidence from this study are consistent with considerable underreporting of pertussis infection in people aged <50 years in the US. Estimates of pertussis incidence based on medical claims for ICD-9-diagnosed pertussis were higher than incidence rates reported by national surveillance, but of a similar order of magnitude. Compared to ICD-9-diagnosed pertussis, estimates of pertussis incidence based on the fraction of cough illness statistically attributable to pertussis were substantially (58–93 times) higher still. The wide variation between the estimated incidence of pertussis obtained using the different methods also emphasizes the challenges in estimating pertussis incidence. However, it is important to keep in mind that the estimates themselves are modeled and should be less construed as reflecting actual incidence than as providing evidence that the majority of cases go undetected, especially with increasing age. These results may aid in the development of public health programs aimed at reducing the burden of pertussis in the US.

## Materials and methods

### Study design

This was a retrospective database cohort study in a population of commercially insured US people aged <50 years with a database claim for ICD-9-coded pertussis or cough illness during the period from 1 January 2008 to 31 December 2013.

### Data sources

Data were obtained from 4 databases: the IMS PharMetrics Plus database of medical insurance claims; the Commercial Outpatient Laboratory (COL) database of laboratory test results; the CDC influenza surveillance database;^31^ and the RSV data came from the CDC's National Respiratory and Enteric Virus Surveillance System (NREVSS) via a data request.

The IMS PharMetrics Plus database contains data on fully adjudicated medical and pharmaceutical claims for over 150 million unique enrollees across the US. It includes information on diagnosis, procedures, prescriptions and inpatient treatment. The database covers 90% of US hospitals, 80% of US doctors, and 85% of large (Fortune 100) companies. Only health plans submitting data for all members are included in the database, ensuring that the data are representative of the national commercially insured population. The database records details of inpatient and outpatient diagnoses coded using ICD-9 codes. ICD-9 is the official system for assigning codes to diagnoses in the US.

The COL database includes laboratory test results collected from a network of over 1,500 laboratories throughout the US. These laboratories conduct approximately 40% of all outpatient laboratory tests in the US.

The CDC influenza surveillance database contains surveillance data collected by 85 World Health Organization (WHO) collaborating laboratories and 60 NREVSS laboratories located throughout the US. These laboratories report weekly data on the number of respiratory samples tested and the number positive for influenza A and influenza B.

The CDC RSV influenza surveillance database collects surveillance data from NREVSS laboratories located throughout the US. It tracks the number of RSV tests performed and the proportion of positive tests. Age information is not included, but the data are assumed to come primarily from pediatric patients.

### Inclusion and exclusion criteria

The inclusion criteria varied between the 3 analyses in the study.

For the analysis based on ICD-9-diagnosed pertussis (method 1), patients were included if they met all the following criteria: at least one medical claim between 1 January 2008 and 31 December 2013 with an ICD-9 code for pertussis (defined as 033.0 [whooping cough due to *B. pertussis*], 033.9 [whooping cough, unspecified organism], or 484.3 [pneumonia in whooping cough]); aged <50 years at the index diagnosis; continuous enrolment for the whole calendar year in which the index pertussis diagnosis occurred and for 3 months before the date of the index diagnosis; for patients aged <1 year, continuous enrolment from the birth month to the end of the calendar year in which the index pertussis diagnosis occurred.

For the proxy pertussis analysis (method 2), patients were included if they met all the following criteria: at least one medical claim between 1 January 2008 and 31 December 2013 with an ICD-9 code for cough illness (defined as 786.2 [cough], 466.0 [acute bronchitis], 466.1 [bronchiolitis], or 487.1 [influenza with other respiratory manifestations]); pertussis laboratory data within 3 months before or after the first cough illness diagnosis date (index date), with a confirmed test result that was either negative (controls) or positive (cases); aged <50 years at the index date; continuous enrolment for at least 3 months before the index date.

For the analysis of cough illness statistically attributable to pertussis (method 3), patients were included if they met all the following criteria: at least one medical claim between 1 January 2008 and 31 December 2013 with an ICD-9 code for pertussis or cough illness (defined as 033.0 [whooping cough due to *B. pertussis*], 033.9 [whooping cough, unspecified organism], or 484.3 [pneumonia in whooping cough], 786.2 [cough]; 466.0 [acute bronchitis]; 466.1 [bronchiolitis], or 487.1 [influenza with other respiratory manifestations]); aged <50 years at the date of the first diagnosis meeting the above criteria.

## Data analysis

### Demographic and clinical characteristics

For the ICD-9-diagnosed pertussis and proxy pertussis analyses (methods 1 and 2), database records were reviewed for 90 days before the index event to identify any comorbid diagnoses and to confirm the absence of previous pertussis diagnoses. Patients aged 18 y or over were assigned a comorbidity score using the Dartmouth-Manitoba modification of the Charlson comorbidity index.^32^

Data were originally categorized into 4 geographic regions: Northeast, Midwest, South, and West. Due to a low sample count in the West region, this was subsequently combined with the Midwest region.

### Projected incidence based on ICD-9-diagnosed pertussis (method 1)

For each of the study years (2008 to 2013), patients with ICD-9-diagnosed pertussis (033.0 [whooping cough due to *B. pertussis*], 033.9 [whooping cough, unspecified organism], or 484.3 [pneumonia in whooping cough]) were identified from the PharMetrics Plus database, stratified by age, gender and geographic region. The total number of enrollees in each of these categories in the database was determined for each calendar year (the ‘eligible count’). The sample of eligible patients was then compared with the national census population of insured individuals in the same age group, gender and geographic region (Northeast, South and Midwest/West), and projection weights calculated using the following formula:

$$Weight(strata)=\frac{Census\;Counts(strata)}{Eligible\;Counts(strata)}$$

These projection weights were then applied to the ICD-9 pertussis cases in the database to estimate the number of pertussis cases nationwide for the insured population. Incidence rates per 100,000 insured persons were calculated by dividing the nationally projected number of pertussis cases by the number of insured persons in the US according to age group and year, obtained from the US Census Bureau's annual Current Population Survey (CPS).^33^

The sensitivity and specificity of the 3 pertussis ICD-9 diagnosis codes were assessed by linking the COL laboratory test data to the PharMetrics Plus medical insurance claims data. Sensitivity was calculated by dividing the number of true positives (i.e., ICD-9 code and positive laboratory test for *B. pertussis*) by the sum of the true positives and false negatives (i.e. no ICD-9 pertussis code but positive laboratory test). Specificity was calculated by dividing true negatives (i.e., no ICD-9 pertussis code and a negative *B. pertussis* laboratory test) by the sum of the true negatives and false positives (i.e. ICD-9 pertussis code but negative laboratory test).

### Projected incidence based on proxy pertussis (method 2)

Clinical events that could indicate an undiagnosed pertussis case were identified from a literature review, and were included as proxy measures of pertussis in building the predictive model. These varied by age group (<1, 1–6, 7–10, 11–18, and 19–49 years) and included symptoms such as cough, acute upper respiratory tract infection, bronchitis/bronchiolitis, dyspnea, fever, nasal/sinus problems, croup, pneumonia asthma, and allergic rhinitis. The final model specification was based on a logistic regression analysis with laboratory pertussis test status (yes/no) as the dependent variable. Independent variables were based on the 10 most frequently observed diagnoses, procedures and drugs (antibiotic and respiratory therapies only) coded within 90 d before the positive pertussis laboratory test. The regressions were conducted separately for each age group, utilizing the combined sample across all the study years. For each age group, predicted probabilities based on the final model were generated to determine the cutoff point for pertussis or non-pertussis. Sensitivity and specificity were calculated.

Once the final model was developed, it was re-run on samples of randomly selected cough illness patients with a ratio of 9 general cough illness patients (controls) to 1 laboratory-confirmed pertussis case. Using the previously determined cutoff point, the proportion of patients with pertussis was estimated for each age group and region. Five hundred bootstrapping samples were generated for each age group to obtain the mean and 95% CI of estimated pertussis incidence by age group and region in the PharMetrics Plus database. These incidence data were then projected to the US national level using the projection weights described above for ICD-9-diagnosed pertussis.

### Projected incidence based on the fraction of cough illness statistically attributable to pertussis (method 3)

To project incidence based on the fraction of cough illness statistically attributable to pertussis, we used an approach similar to that used in an earlier analysis conducted in people aged ≥50 years,^21^ modified to include influenza and RSV data (in order to reduce the proportion of unattributed cough illness). Projected monthly incidence rates for ICD-9-diagnosed cough illness (033.0 [whooping cough due to *B. pertussis*], 033.9 [whooping cough, unspecified organism], 484.3 [pneumonia in whooping cough], 786.2 [cough], 466.0 [acute bronchitis], 466.1 [bronchiolitis], or 487.1 [influenza with other respiratory manifestations]) were calculated and used as the dependent variable in the regression model. A monthly time series of positive laboratory tests (for influenza, pertussis, RSV) were used to quantify the incidence of medically attended cough illness attributed to *B. pertussis*. As the COL database included only outpatient tests and infants with pertussis are more likely to be hospitalized, an inpatient discharge diagnosis of pertussis was used as a proxy for laboratory-confirmed pertussis in the group aged <1 year.

The regression model quantified the relationship between the monthly variation in cough illness diagnoses and the monthly variation in positive laboratory tests for each of the pathogens included (pertussis, influenza and RSV).

The model is described as follows:

y =β0 + β1*(B. pert)+β2*(RSV)+β3*(Influenza)+β4*sine(2*t*π/12)+β5*cosine(2*t*π/12)+β6*t+β7*(t2) + e

where: y = cough illness incidence (projected); β0 = constant term; B. pert = laboratory tests positive for *B. pertussis* (monthly data) and inpatient infant proxy lab counts (<1 model only); β1 = regression coefficient used for estimating the number of cough illness events attributable to *B. pertussis*; RSV = laboratory tests positive for RSV; Β2 = regression coefficient used for estimating the number of cough illness events attributable to RSV; Influenza = laboratory tests positive for influenza (monthly data); Β3 = regression coefficient used for estimating the number of cough illness events attributable to influenza; β4*sine(2*t*π/12) and β5*cosine(2*t*π/12) = regression coefficients and their associated terms to account for seasonal changes in the dependent variable (y); β6*t and β7*(t2) = regression coefficients and their associated terms to account for linear and quadratic time trends in the dependent variable (y); e = error term.

Models were run by age group and geographic region. Negative pathogen model terms were removed from subsequent models until a final model was obtained. This analysis produced a regression coefficient indicating the amount of the incidence of cough illness that was attributed to each of the independent variables. The pertussis coefficient was then multiplied by the value of the independent variable for *B. pertussis* in the observed data (the number of positive laboratory tests in a given month), to estimate the monthly incidence of cough illness attributed to *B. pertussis*. These monthly rates were summed for a 12-month period to yield annual rates of cough illness attributed to *B. pertussis*. The annual incidence rates were reported by geographic region for each year of the study (no valid age-specific models could be developed). These incidence data were then projected to the US national level using the projection weights described above for ICD-9-diagnosed pertussis.

## Abbreviations

ACIP: Advisory Committee on Immunization Practices
CDC: Centers for Disease Control and Prevention
CI: confidence interval
COL: Commercial Outpatient Laboratory
DTaP: diphtheria, tetanus and acellular pertussis
DTP: diphtheria-tetanus-pertussis
ICD-9: International Classification of Diseases, Ninth Revision, Clinical Modification
NREVSS: National Respiratory and Enteric Virus Surveillance System
PCR: polymerase chain reaction
RSV: respiratory syncytial virus
Tdap: tetanus, diphtheria and acellular pertussis
US: United States
WHO: World Health Organization
